# Excess of Methyl Donor in the Perinatal Period Reduces Postnatal Leptin Secretion in Rat and Interacts with the Effect of Protein Content in Diet

**DOI:** 10.1371/journal.pone.0068268

**Published:** 2013-07-01

**Authors:** Fanny Giudicelli, Anne-Laure Brabant, Isabelle Grit, Patricia Parnet, Valérie Amarger

**Affiliations:** 1 INRA, UMR1280, Physiologie des Adaptations Nutritionnelles, Nantes, France; 2 Université de Nantes, UMR 1280, Physiologie des Adaptations Nutritionnelles, Nantes, France; State University of Rio de Janeiro, Biomedical Center, Institute of Biology, Brazil

## Abstract

Methionine, folic acid, betaine and choline interact in the one-carbon metabolism which provides methyl groups for methylation reactions. An optimal intake of these nutrients during pregnancy is required for successful completion of fetal development and evidence is growing that they could be involved in metabolic long-term programming. However, the biological pathways involved in the action of these nutrients are still poorly known. This study investigated the interaction between methyl donors and protein content in maternal diet during the preconceptual, pregnancy and lactation periods and the consequences on the rat offspring in the short and long term. Methyl donor supplementation reduced leptin secretion in offspring, whereas insulin levels were mostly affected by protein restriction. The joint effect of protein restriction and methyl donor excess strongly impaired postnatal growth in both gender and long term weight gain in male offspring only, without affecting food intake. In addition, rats born from protein restricted and methyl donor supplemented dams gained less weight when fed a hypercaloric diet. Methylation of the leptin gene promoter in adipose tissue was increased in methyl donor supplemented groups but not affected by protein restriction only. These results suggest that maternal methyl donor supplementation may influence energy homeostasis in a gender-dependent manner, without affecting food intake. Moreover, we showed that macronutrients and micronutrients in maternal diet interact to influence the programming of the offspring.

## Introduction

It is now widely accepted that maternal nutrition in the periconceptual and perinatal periods, besides providing nutrients required for an optimal fetal growth and development may participate in the programming of metabolic disorders such as hypertension, impaired glucose tolerance, dyslipidemia and overweight [1–6].

Maternal protein restriction (PR) is a commonly used model of nutritional programming which impacts on fetal and postnatal growth and long term metabolism [7–10]. The precise mechanisms underlying these effects are still unclear, probably because of their large number and variety, from amino acid and nitrogen shortage to tissue structural alterations and impaired gene expression [11–15]. The effect of maternal PR on epigenetic regulation of several genes in various tissues was demonstrated [16–20] and epigenetic mechanisms are now recognized as major contributors to the influence of early environment on gene expression in the long term [21]. Several nutrients were shown to be specifically important in the regulation of these mechanisms. For instance, the adverse effects of protein restriction on DNA methylation can be reversed by the addition of a supplement of folic acid [22–24] and maternal choline deficiency was associated with alterations of epigenetic regulation [25–27].

Besides these effects observed on experimental models, many clinical observations argue in favour of multiple benefits of folic acid supplementation on pregnancy outcomes [28–30], and supplementation with multiple micro nutrients and vitamins appear even more beneficial in developing countries [31]. An adequate maternal supply in folate and choline is required for an optimal neuro-development [32] and evidence is growing that these nutrients may exert a programming effect on both metabolic and cognitive outcomes [25,33]. Folic acid, choline and methionine participate in multiple metabolic pathways among which nucleotide and protein synthesis, cell membrane biosynthesis and neurogenesis [34]. They are all involved in the one-carbon metabolic pathway which plays a major role in fetal development and programming. This pathway controls the synthesis and degradation of homocysteine and provides methyl groups for methylation reactions, in particular those associated with the epigenetic control of genome integrity and regulation. Many studies have investigated the effects of a deficiency in folic acid and related methyl donors [27,32,35] and it is widely admitted that deleterious effects are associated with a lack of these nutrients, although the underlying mechanisms are still unclear, probably because of the large panel of action of these nutrients.

In this work, we addressed the issue of the interaction between protein restriction and methyl donor supply in maternal diet and the consequences on fetal, postnatal and long term growth. We made the choice to supplement the maternal diet with an excess of several nutrients from the one-carbon metabolic pathway (methionine, choline, folic acid, Vit B12, zinc and betaine) in order to create a nutritional epigenetic challenge by influencing the availability of methyl groups [36,37]. We present here the consequences on leptin and insulin secretion, early growth and long term weight gain.

## Materials and Methods

### Ethics statement

Animal procedures and maintenance were conducted in accordance with the European Communities Council Directive 2007/526/CE and were approved by the Institut National de la Recherche Agronomique (INRA, Paris, France). The protocol was approved by the local ethics committee for animal experimentation (Comité Régional d’Ethique en Expérimentation Animale Pays de Loire under the license number CEEA.2010.02).

### Animals and diets

Thirty two virgin female Sprague Dawley rats, 7 weeks old, weighing 200-220g, were purchased from Janvier (Le Genest Saint Isle, France), and housed individually on a 12h: 12h light: dark cycle, with free access to food and water. Upon arrival, they were randomized to receive one of the following diets (n=8 per group): (a) control (C) diet, (b) control diet supplemented with methyl donors (MD) (Csup), (c) protein restricted diet (R) or (d) protein restricted diet supplemented with MD (Rsup). Diets were purchased from Arie Blok, Woerden, the Netherlands (composition in Table S1). We decided to supplement maternal diet with various MD and cofactors (methionine, folic acid, choline, betaine, zinc, Vit B12) in order to increase methyl metabolism and subsequently influence the epigenetic regulation of epilabile loci [36,37]. Females were fed these diets for 21 to 28 days before mating, and throughout gestation and lactation. After overnight housing with males, mating was confirmed by the presence of spermatozoa in vaginal smears. At birth, litters were culled to 8 pups per dam (4 males and 4 females). The weight, food intake of pregnant dams and pups weight were recorded daily. At birth (D0) or weaning (D21) (after overnight fasting for D21), subsets of pups were sacrificed by decapitation and tissues (adipose tissue, liver) quickly dissected, snap frozen in liquid nitrogen and stored at -80°C. Plasma was obtained from whole blood collected on EDTA or heparinized tubes followed by centrifugation at 2500g for 15min at 4°C.

The offspring were weaned at 21 days of age onto the same standard growth diet until the age of 42 days (SAFE A03) and fed a standard maintenance (SAFE A04) diet until the age of 23 weeks. At 23 weeks, all animals were given free access to a hypercaloric high-fat high-sucrose western diet (4021.87, Arie Blok, Woerden, the Netherlands) for 4 weeks and sacrificed after an overnight fasting. Animal weight and food intake were recorded three times a week.

### Plasma hormones and metabolites determination

Plasma cholesterol, glucose and triglyceride (TG) levels were determined using commercial kits (Cholesterol RTU, Glucose RTU, TG PAP 150) from bioMérieux (Marcy l’étoile, France). Leptin and insulin concentrations were determined with specific ELISA kits (Rat Leptin and Rat/Mouse insulin 96-well plate assay) from Millipore.

### Real time quantitative RT-PCR

Total RNA was extracted from white adipose tissue using Qiazol (Qiagen Sciences, Maryland, USA) and quantified using the Nanovue spectrophotometer (GE Health Care, France). RNA integrity was confirmed by agarose gel electrophoresis and, for a random set of samples, using the Agilent BioAnalyser 2100. cDNA synthesis and Real Time PCR were performed as previously described [38]. Two reference genes were used as internal control, β-actin and Beta 2 Microglobulin (B2M). They were chosen and validated using the Genorm® Software [39]. Relative mRNA expression was calculated using the 2^-∆∆Ct^ method. Primer sequences are given in Table S2.

### Pyrosequencing DNA methylation analysis

Genomic DNA was extracted from white adipose tissue using the Nucleospin® Tissue kit (Macherey Nagel, GmbH & Co, France). Two micrograms were submitted to bisulfite modification using the Methyl Detector bisulfite modification kit (Active Motif, Europe, Rixensart, Belgium) according to the manufacturer’s instructions. Two µl of the modified DNA was amplified by nested PCR with primers designed using MethPrimer [40], using the Pyromark PCR Kit (Qiagen Sciences, Maryland, USA) under the following conditions 95°C-10 min, 25 cycles (95°C-30s, 56°C-1min 30s, 72°C-1min) for the first PCR and 95°C-10 min, 30 cycles (95°C-30s, 56°C-30s, 72°C-30s) for the second PCR. Two µl from the 1^st^ PCR were used as template for the second PCR. Sample preparation and pyrosequencing were performed using the Pyromark Q96 instrument (Qiagen) following the manufacturer’s recommendations. All samples (6 to 8 individuals/group) were analysed in duplicates in separate experiments and the obtained values were averaged. Primer sequences are given in Supplementary Table S2.

### Statistical analyses

Data were analysed using GraphPad Prism® 5 (GraphPad software Inc., La Jolla, CA, USA). Comparison of data within the four experimental groups was performed using the Kruskall-Wallis test followed by Dunn’s multiple comparison test because variances differed between groups and/or n<10/group. Mann Whitney test was used when only two groups were compared. Interaction between MD and protein content in maternal diet or between gender and diet was tested using two-way ANOVA, followed by Bonferroni’s post-test, which adjusts for multiple testing. P<0.05 was considered significant. Correlations were tested using the non-parametric Spearman’s test.

## Results

### Maternal characteristics

Whereas all dams had similar body weight at the beginning of the experiment, dams fed the MD supplemented diets weighed less at the end of the habituation period, particularly the Rsup group (Table 1). Mating was confirmed for 6, 7, 6 and 7 females for the C, Csup, R and Rsup groups, respectively and all females were indeed pregnant except for 3 females in the Csup group. Total food intake during gestation was reduced by 20 to 25% in the Rsup and Csup compared to the R and C dams, respectively, and body weight at the end of gestation was correlated to food intake (r=0.75, p<0.0001). The number of pups per litter varied from 8 to 17, and litters from the Csup group were significantly smaller than litters from the control group (Table 1).

**Table 1:** Litter size, body weight and total food intake of dams during gestation and lactation.

	C n=6-5*	Csup n=4-3*	R n=6-5*	Rsup n=7-6*
**Body weight G0 (g)**	269.2±3.9^a^	246.8±4.2^ab^	266.4±4.0^a^	239.3±5.0^b^
**Body weight G20 (g)**	432.7±12.1^a^	359.9±5.1^b^	397.9±5.0^ab^	356.7±7.0^b^
**Body weight D21 (g)**	337.9±11.7^a^	293.9±7.5^ab^	278.0±3.9^ab^	228.5±9.0^b^
**Cumulative food intake during gestation (kcal)**	1512±65^ac^	1113±21^b^	1566±36^a^	1255±51^bc^
**Cumulative food intake during lactation (kcal)**	2732±111^a^	2256±59^a^	2498±59^a^	1569±80^b^
**Litter size**	14.3±0.8^a^	10.7±0.9^b^	13.1±0.5^ab^	12.7±0.1^ab^

* n number of dams during gestation-lactation

(G0 = first day of gestation, G20 = last day of gestation and D21 = last day of lactation) (Mean±SEM).

^a b c^ Groups that share the same superscript are not different from each other (p<0.05, Kruskal-Wallis and Dunn’s post hoc test). C: Control, Csup: Control MD supplemented, R: Protein Restricted, Rsup: Protein Restricted MD supplemented

Food intake during lactation remained lower for Rsup dams, but increased throughout lactation for the Csup dams, leading to a cumulative intake similar to controls (Table 1).

### Impact on birth weight and postnatal growth

Surprisingly, pups born from dams fed the PR diet (R) were slightly heavier at birth than pups from control mothers (males +6%, p<0.05, females +5%, ns) (Table 2). MD supplementation was associated with a lower birth weight, mostly in the Rsup group compared to the R group (males -12% and females -11%, p<0.0001), and in the Csup group compared to the C group for the females (-6%, p<0.05) (Table 2). Data analysis using two-way ANOVA revealed an interaction between MD and protein content (p=0.0002 for males and p=0.04 for females) on birth weight.

**Table 2:** Offspring body weight at birth and weaning, and metabolic and hormonal parameters at weaning.

	**C**	**Csup**	**R**	**Rsup**	
**Birth weight (g)**					
**Males (n)**	6.5±0.1 (29)^a^	6.3±0.05 (21)^a,c^	6.9±0.06 (45)^b^	6.1±0.07 (40)^c^	
**Females (n)**	6.2±0.07 (40)^a^	5.8±0.09 (22)^b^	6.5±0.06 (35)^a^	5.8±0.07 (49)^b^	
**Weight at weaning (g)**					
**Males (n)**	46.8±1.1 (17)^a^	40.5±1.6 (12)^ab^	37.1±0.7 (20)^b^	26.2±0.6 (24)^c^	
**Females (n)**	46.6±0.8 (14)^a^	39.8±1.7 (12)^ab^	36.5±0.8 (19)^b^	25.9±0.6 (24)^c^	
**Glucose (g/l) – Day 21**					
**Males (n)**	0.97±0.01 (8)^a^	1.04±0.03 (6)^ab^	0.96±0.02 (8)^ab^		0.84±0.02 (8)^b^
**Females (n)**	1.04±0.02 (8)^a^	1.23±0.03 (6)^b^	1.17±0.06 (8)^b^		1.02±0.02 (8)^ab^
**Triglycerides (g/l) – Day 21**					
**Males (n)**	1.97±0.20 (8)^a^	1.26±0.16 (6)^b^	0.56±0.04 (8)^c^		0.50±0.04 (8)^c^
**Females (n)**	2.08±0.25 (8)^a^	1.16±0.22 (6)^b^	0.65±0.07 (8)^c^		0.51±0.04 (8)^c^
**Cholesterol (g/l) – Day 21**					
**Males (n)**	2.15±0.18 (8)^a^	1.80±0.13 (6)^ab^	1.83±0.12 (8)^ab^		1.77±0.07 (8)^b^
**Females (n)**	2.04±0.13 (8)^a^	1.66±0.11 (6)^a^	1.69±0.07 (8)^a^		1.89±0.10 (8)^a^
**Insulin (ng/ml) – Day 21**					
**Males (n)**	0.63±0.04 (8)^a^	0.65±0.09 (6)^a^	0.34±0.04 (8)^b^		0.18±0.03 (8)^b^
**Females (n)**	0.69±0.12 (8)^a^	0.70±0.07 (6)^a^	0.44±0.05 (8)^b^		0.24±0.02 (8)^b^
**Leptin (ng/ml) – Day 21**					
**Males (n)**	3.26±0.33 (8)^a^	1.85±0.24 (6)^b^	4.16±0.49 (8)^a^		1.23±0.17 (8)^b^
**Females (n)**	4.01±0.53 (8)^a^	2.43±0.19 (6)^b^	4.23±0.65 (8)^a^		0.81±0.16 (8)^c^
**Dams (n)**	3.58±0.73 (5)^a^	3.05±0.59 (3)^a^	2.53±0.39 (5)^ab^		1.20±0.00 (6)^b^

(Mean±SEM). ^a b c^ Groups that share the same superscript are not significantly different from each other (p<0.05, Kruskal-Wallis and Dunn’s post hoc test, independently for each gender).

C: Control, Csup: Control MD supplemented, R: Protein Restricted, Rsup: Protein Restricted MD supplemented

Postnatal growth was dramatically impaired in the protein restricted groups (R and Rsup) compared to controls with Rsup and R pups weighing about 45% and 21% less than controls at weaning, respectively (Table 2).

### Plasma metabolic and hormonal parameters at D21

Plasma glucose was reduced by about 25% in Rsup males and increased in Csup and R females (+18 and 12% respectively) compared to controls. Cholesterol was reduced in Rsup males (-18%) as well, whereas there was no difference between groups for females. Plasma triglyceride levels were markedly reduced in the three experimental groups compared to controls, particularly in the R and Rsup groups whose concentrations were about 4 times less (Table 2).

Pups from the protein restricted groups (R and Rsup) had lower insulin levels, (Table 2). This effect was emphasized by the MD supplementation (Rsup group, -70% compared to controls), whereas MD supplementation in the absence of protein restriction had no effect (Csup).

Plasma leptin at D21 was strongly reduced (-40 to -70%) in the MD supplemented groups (Csup and Rsup) in both males and females as compared to controls. A strong cumulative effect of both protein restriction and MD supplementation (Rsup group) was observed in females which showed leptin levels 5 times lower than controls (Table 2). In order to test if pups plasma leptin was influenced by maternal leptin through milk intake, we measured the level of plasma leptin in lactating dams just before weaning. There was no significant difference between C, Csup and R dams but a 3 times lower level in Rsup dams compared to controls (Table 2). Dams plasma leptin was well correlated with body weight (r=0.8 p<0.0001) in all groups.

### Leptin gene expression and promoter DNA methylation

**Figure 1:**
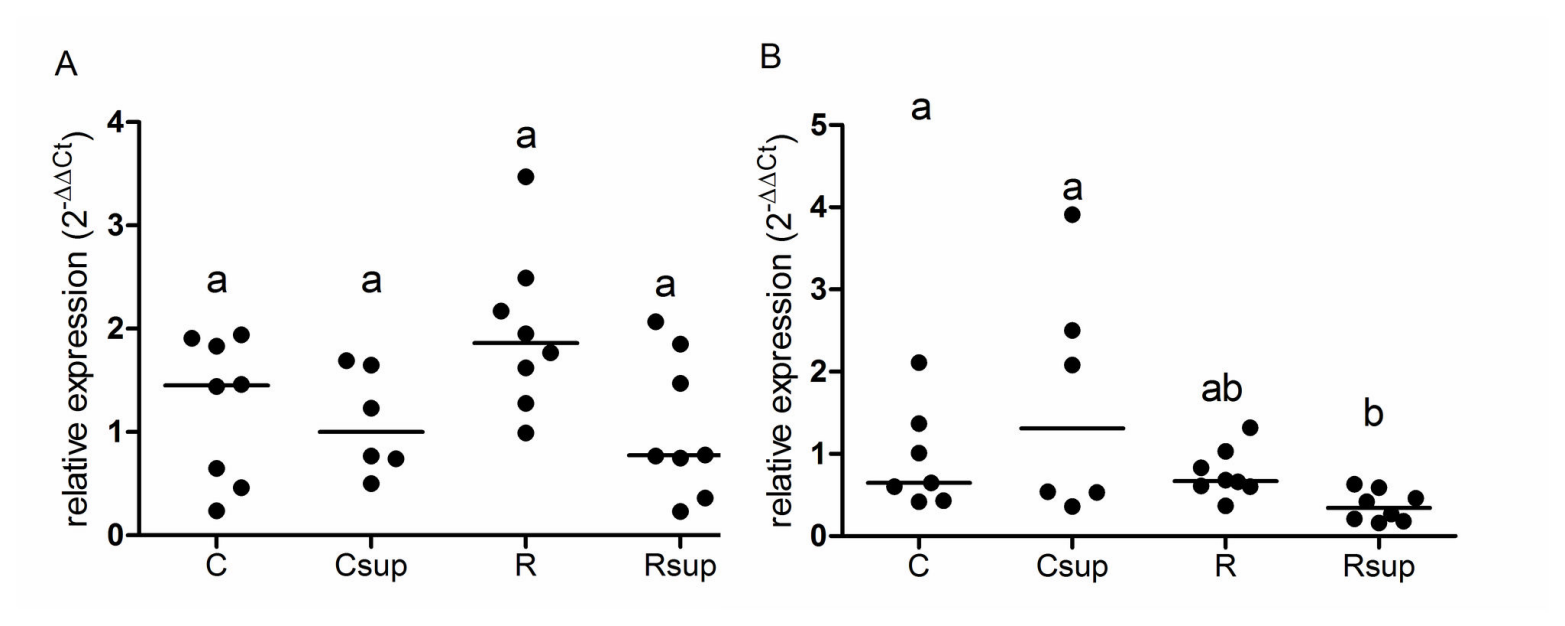
Relative expression of the Leptin gene in adipose tissue at D21. Leptin gene expression was measured on white adipose tissue from males (A) and females (B) sacrificed at D21 after overnight fasting. Individual data are presented using a scatter plot with median. ^a,b^Groups that share the same superscript are not significantly different from each other (p<0.05, Kruskal-Wallis and Dunn’s post hoc test). C: Control, Csup: Control MD supplemented, R: Protein Restricted, Rsup: Protein Restricted MD supplemented.

Because of the large variations observed in offspring plasma leptin, we tested whether this might be linked to changes in the expression level of the Lep gene in adipose tissue. Surprisingly, we did not show any evidence of significant changes that may explain the observed variations in plasma leptin although Rsup males had a slightly lower expression level than controls (p<0.05 Dunn’s test) (Figure 1).

**Figure 2:**
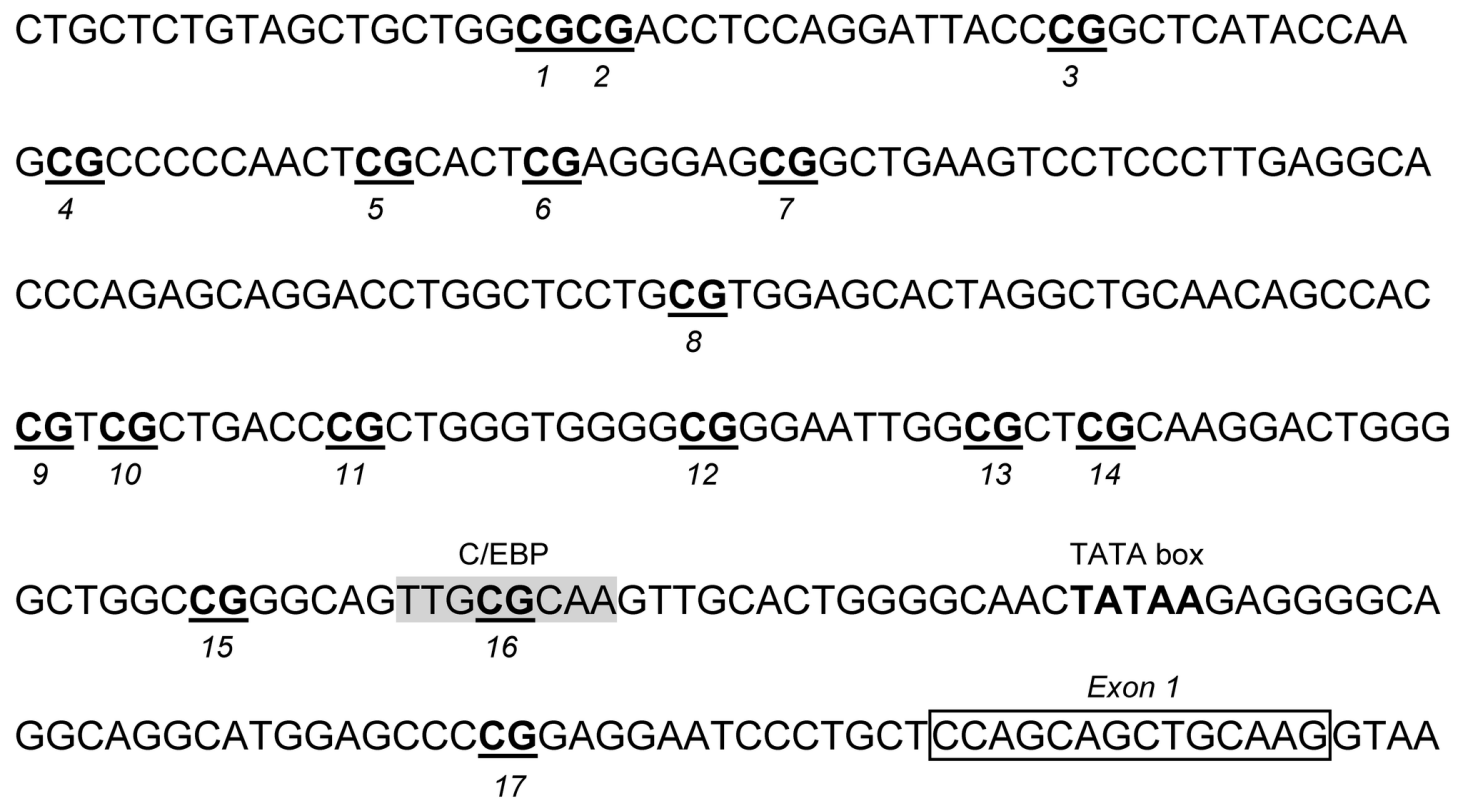
Nucleotide sequence of the rat leptin gene promoter. CpG sites which methylation level was analysed are numbered from 1 to 16. Positions of the C/EBP transcription factor binding site, the TATA box and the exon 1 are indicated. C: Control, Csup: Control MD supplemented, R: Protein Restricted, Rsup: Protein Restricted MD supplemented.

**Figure 3:**
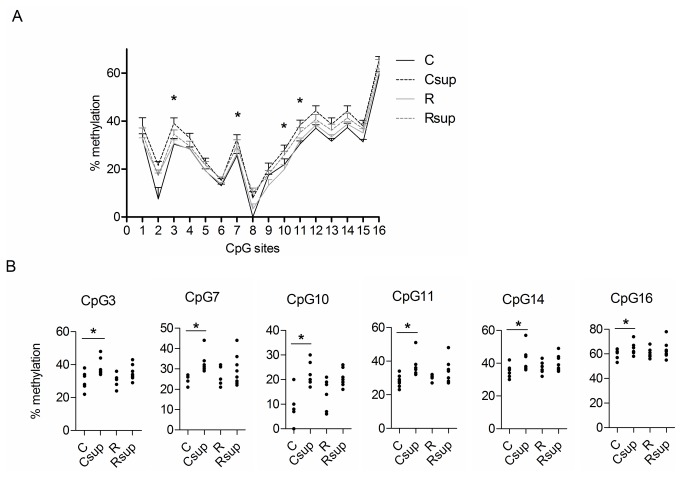
Leptin gene promoter methylation in male offspring at D21. (A) Methylation level at CpG sites 1 to 16 in each group of animals was measured by pyrosequencing on genomic DNA extracted from white adipose tissue. Data are presented as mean±SEM for 6 to 8 samples per group (* p<0.05 Kruskal Wallis test). (B) Methylation levels at individual CpG sites (*p<0.05 Mann Whitney test between C and Csup groups). C: Control, Csup: Control MD supplemented, R: Protein Restricted, Rsup: Protein Restricted MD supplemented.

Since we observed a strong effect of maternal MD supplementation on offspring leptin levels, we analysed the DNA methylation of the leptin gene promoter. The region spanning 282 bp upstream the transcription start site (Figure 2) contains 16 CpG sites which were analysed by pyrosequencing on genomic DNA extracted from white adipose tissue of 21 days old rats. The methylation level over this region harbored a great heterogeneity (from 0 to about 60% from one CpG site to another) (Figure 3A). Methylation levels were overall higher in the two methyl supplemented groups (Csup and Rsup) compared to the unsupplemented groups (C and R) and the control group displayed the lowest methylation level at all CpG sites. Significant differences over the four groups (Kruskall Wallis test, p<0.05) were observed for CpG sites 3, 7, 10 and 11, and pairwise comparison between C and Csup groups was significant for CpG sites 3, 4, 6, 7, 10, 11, 12, 13, 14 and 16 (Mann Whitney p<0.05). Only CpG 10 was significantly less methylated in Rsup than controls (p=0.01). Inter-individual variability was observed for most CpG sites, as evidenced on the scatter plot graphs (Figure 3B). CpG sites 8 and 2 were completely unmethylated for all (CpG 8) or 2 out of 6 (CpG2) individuals from the control group whereas they had methylation level ranging from 0 to 20% (CpG8) or 10 to 30% (CpG2) in the other groups (data not shown). The CpG site 16 is situated in a C/EBP transcription factor binding site, which is highly conserved between species [41] and which methylation status was shown to strongly influence the expression level of the gene [42]. This CpG was highly methylated in our samples (58% on average in the C group to 65% in the Csup group), compare to the other CpG sites in the region.

### Long term growth, food intake and metabolic parameters

Differences in body weight that were observed at weaning disappeared for females during the month following weaning and this situation lasted until week 22 (Table 2).

Control males kept a higher body weight that the 3 experimental groups throughout the whole experiment, but the difference was significant only with Rsup males which were on average 17% lighter than controls from weaning until week 22 (p<0.05). Csup males tend to gain less weight from the age of 10 weeks and, from that point, their growth trajectory was similar to that of males from the R group. From the age of 23 weeks, although the differences were not statistically significant, Csup males tend to weigh less than R animals.

Relative food intake was on average 20% higher (p=0.0002) for Rsup males compare to C males during the week following weaning, and an intermediate situation for Csup and R animals (data not shown). However, these differences tend to disappear and, by 6 weeks of age, there was no more difference between the four groups. Female food intake was only slightly higher in the Rsup group compare to the C group (+9%, p=0.01) at 4 weeks and there was no difference between groups from 6 weeks of age.

**Figure 4:**
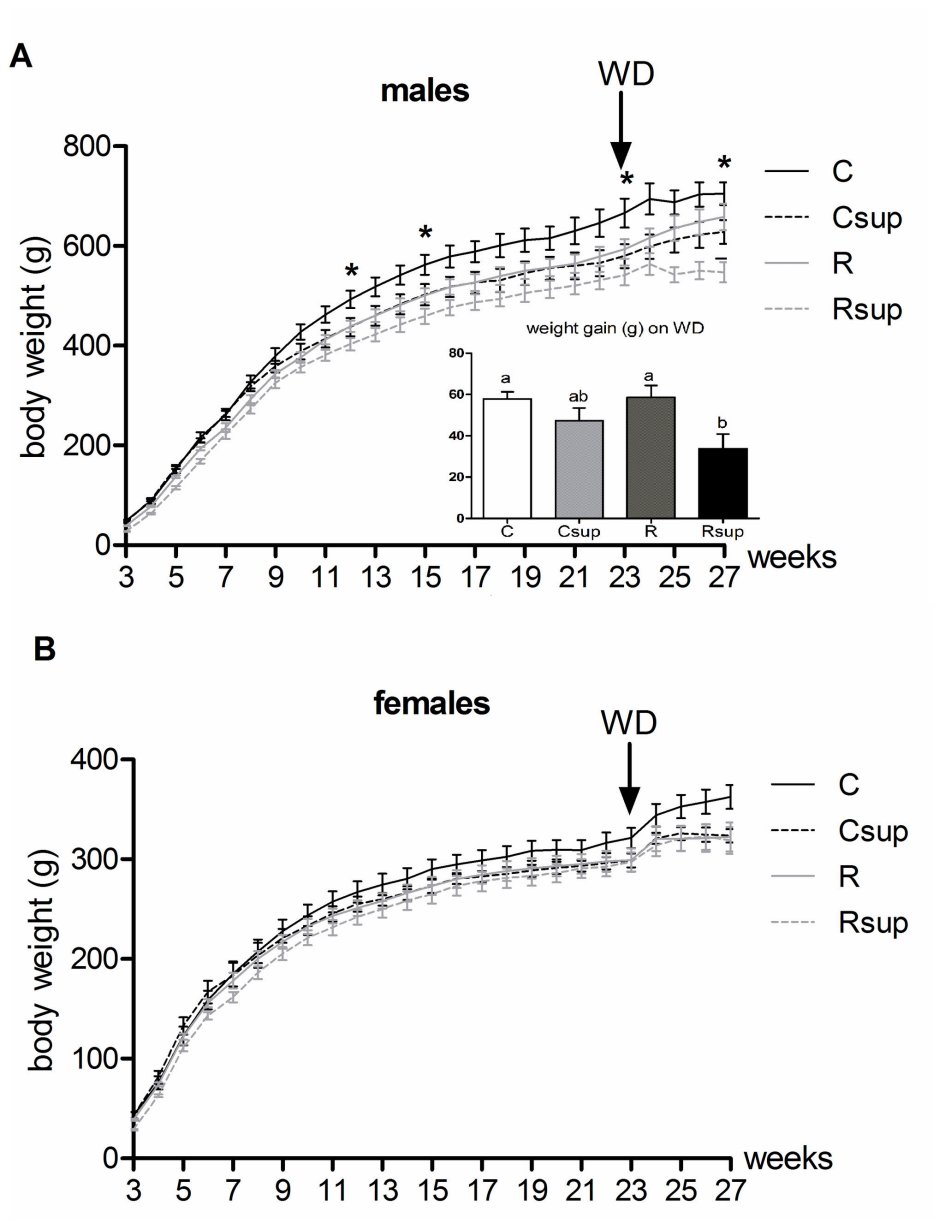
Offspring growth from week 3 to 27. Animals were fed chow diet until week 23 and western diet (WD) from week 23 to 27. Body weight for males (A) and females (B) were recorded three times a week. Values are expressed as mean±SEM (* p<0.05 Kruskal Wallis test). Male weight gain on WD corresponds to weight difference between week 23 and week 27 (mean±SEM). ^a,b^Groups that share the same superscript are not significantly different from each other (p<0.05, Kruskal-Wallis and Dunn’s post hoc test). C: Control, Csup: Control MD supplemented, R: Protein Restricted, Rsup: Protein Restricted MD supplemented.

When given free access to WD, all animals increased their food intake during the first three days and adjust it thereafter. After 4 weeks of WD, energy intake was about 20% higher than previous intake under regular chow diet. The consequence was a similar weight gain of about 10 to 15% for all females of the four groups (Table 3). Weight gain in males varied from 6 to 10% and was significantly lower in the Rsup group compared to C and R groups. Therefore, at the end of the WD period, the difference between Rsup and control males was even higher than before (-32%, p<0.01) (Figure 4).

**Table 3:** Body weight at 23 and 27 weeks.

	**C**	**Csup**	**R**	**Rsup**
**Weight at 23 weeks (g)**				
**Males (n)**	646±27 (8)^a^	565±26 (4)^ab^	578±19 (8)^ab^	530±17 (8)^b^
**Females (n)**	309±10 (8)^a^	293±5 (4)^a^	295±9 (8)^a^	290±9 (8)^a^
**Weight at 27 weeks (g)**				
**Males (n)**	704±22 (4)^a^	623±23 (4)^ab^	658±26 (6)^ab^	551±17 (6)^b^
**Females (n)**	362±12 (8)^a^	323±6 (4)^a^	324±14 (8)^a^	318±13 (8)^a^

(Mean±SEM). ^a b^ Groups that share the same superscript are not significantly different from each other (p<0.05, Kruskal-Wallis and Dunn’s post hoc test). C: Control, Csup: Control MD supplemented, R: Protein Restricted, Rsup: Protein Restricted MD supplemented.

**Figure 5:**
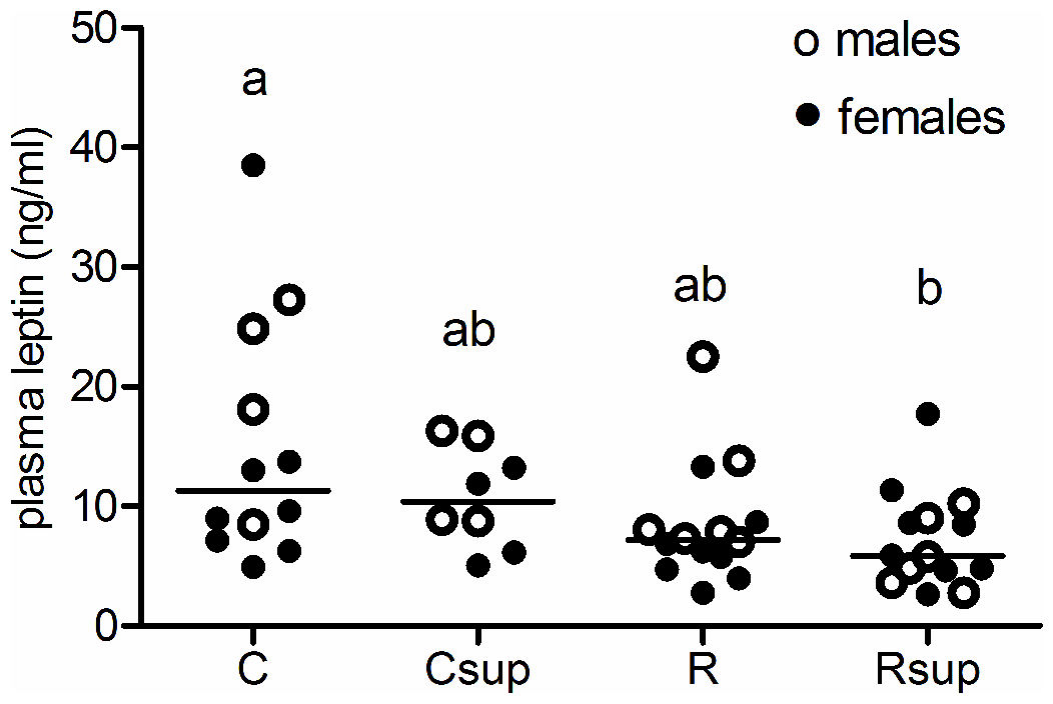
Plasma leptin in 27 week old rats. Plasma leptin was measured on blood sampled on animals sacrificed after overnight fasting (n=4 to 6 per gender per group). ^a,b^Groups that share the same superscript are not significantly different from each other (p<0.05, Kruskal-Wallis and Dunn’s post hoc test). C: Control, Csup: Control MD supplemented, R: Protein Restricted, Rsup: Protein Restricted MD supplemented.

Plasma leptin was still reduced in the Rsup group compared to controls in both gender (p<0.05) but not anymore in the Csup group (Figure 5). Plasma leptin was correlated with body weight for females (r=0.8, p<0.0001), but weakly for males (r=0.59, p=0.005). There was no difference between groups in leptin gene expression (data not shown).

There was no significant difference between gender and between groups for plasma triglycerides, cholesterol, glucose and insulin at the age of 27 weeks (data not shown).

## Discussion

We observed here that maternal methyl donor supplementation was associated with lower leptin levels in the offspring at weaning, which, to our knowledge, has never been reported before. Reduced or delayed leptin surge, which occurs normally during the second week of life in rodents, was observed in models of maternal under nutrition and/or protein restriction and was associated with postnatal growth restriction [16,43]. In our model, postnatal growth was impaired in the protein restricted groups compared to controls, but plasma leptin was predominantly reduced in both MD supplemented groups suggesting an impact of the maternal diet composition itself rather than the growth rate. The presence of MD supplements in the diet therefore seemed to impact the secretion of leptin in the Csup pups whereas there was probably a cumulative effect of the MD and the reduced maternal food intake during lactation for the Rsup pups, as observed in models of maternal caloric restriction [11,44].

In this experiment, we did not measure leptin levels at different stages of pup development, therefore we do not know if the leptin surge occurred or not and when. However, in their model of maternal caloric restriction, Delahaye et al. [11] have shown that a reduced leptin level at D21 is reminiscent of an impaired leptin secretion throughout the suckling period whereas the occurrence of a premature or delayed leptin surge is no longer visible at weaning [38]. We can therefore suggest that the animals from the MD supplemented groups had lower leptin levels from birth until weaning and even persisted low at adulthood for the Rsup.

The way methyl donors content in maternal diet influenced offspring plasma leptin at weaning remains to be determined. Each of the nutrients involved in one-carbon metabolism participates in other biological pathways and we cannot exclude that the observed effects may be due to one of these nutrients rather than the combination of all of them. An excess of zinc, for instance, may be responsible for a reduced maternal nonembryonic weight gain [45]. However, all these nutrients have been shown to be associated with the epigenetic machinery [25–27,46] and epigenetic mechanisms are now admitted to play a major role in the process of nutritional programming [21]. Supplementation with an excess of methyl donors around conception and during gestation is known to induce hypermethylation at epi-labile *loci* [36,37,47] and may be involved in the protection against obesity [48]. We observed a higher methylation level of the leptin promoter gene in the adipose tissue of offspring from the MD supplemented dams. Are these rather moderate effects responsible for the large variations in plasma leptin is questionable. Although we found that the methylation status of the leptin gene promoter was influenced by maternal diet, we did not find evidence of large variations in the expression of the gene that could explain the variation in plasma concentration. However, plasma leptin at a given time point may not perfectly correlate with the expression level of the gene but rather reflect the amount of mature adipocytes. Indeed, the promoter methylation may also reflect a lower differentiation level of the adipose tissue. The leptin gene is highly methylated in undifferenciated pre-adipocytes and undergoes demethylation during the differenciation process [42] allowing the activation of its expression by several transcription factors. The C/EBPα transcription factor plays a major role in the activation of adipocyte-specific genes, among which the leptin gene. It binds to a specific site at position -61 in the leptin gene promoter and its binding is impaired by methylation of the CpG embedded in the binding site. Surprisingly, this CpG site is the one displaying the highest level of methylation (around 60%) in our experiment (CpG n° 16), and other studies [18,49].

Further work is needed to establish if MD supplementation influenced the number of differenciated adipocytes. Since insulin is known to induce pre-adipocyte differentiation [50], low insulin level in the Rsup animals may have accentuated this effect. This would fit well with a probable lower fat mass at that period and is in agreement with the observed lower weight gain at adulthood and lower plasma leptin at 27 weeks of age. Moreover, the fact that Rsup animals had a similar food intake compared to the other groups but display a 20% lower body weight than control rats strongly suggests that they have a different metabolic regulation or energy expenditure. It is now admitted that appropriate leptin levels during neonatal life are required for normal energy balance regulation and hypothalamic development and function [51], and leptin provided through breast milk was shown to protect against several outcomes related to metabolic syndrome [52]. Surprisingly, in our study, pups from the MD supplemented dams combine lower plasma leptin levels at weaning and a reduced diet-induced weight gain at adulthood. Interestingly, the phenotype we observed here on the male offspring is somehow, but to a less extent, similar to the phenotype of the Bhmt (Betaine-homocysteine methyl transferase) knock-out mice model which is characterised by an increased energy expenditure, reduced weight gain and reduced adiposity despite a normal food intake, associated with an hyperhomocysteinemia [53]. In that model, the homocysteine pathway is impaired in liver, where the Bhmt gene is normally expressed, but the whole energy metabolism is disrupted, which shows evidence of the implication of the one-carbon metabolic pathway in the control of energy homeostasis.

In summary, we show here that maternal supplementation with an excess of methyl donors strongly reduced early leptin secretion. When combined with protein restriction which impaired insulin secretion, this supplementation affects long term weight gain in males independently of food intake, which suggest an impact on energy homeostasis and expenditure regulation.

The data presented here are not sufficient to conclude whether maternal MD supplementation is beneficial or deleterious to the offspring submitted to an early carence in protein. However, the observed tendency to a protection against the obesogenic effect of a hypercaloric diet is a very intriguing result. This interesting observation needs to be completed by mechanistic investigation particularly on adipose tissue development and leptin action. Moreover, we observed a maternal hyperhomocysteinemia induced by the MD supplementation (data not shown) that could be of importance since it may impact on several pregnancy outcomes [30]. In regard to these new observations, and although the amount of methyl donors used in our experiment was higher than what is provided through multivitamins dietary supplements, it raises the question of the potential interaction between methyl donors and protein content in maternal diet when prescribing these supplements to pregnant women. Further work is needed to deepen our understanding of micro- and macro-nutriments interactions during such critical periods as pre-conception, pregnancy and lactation.

## Supporting Information

Table S1 Composition of the diets. (DOCX)

Table S2 Sequence of primers used for Real Time qPCR, Nested Bisulfite PCR and pyrosequencing. (DOCX)
