# Supplementary material for: Excess of Methyl Donor in the Perinatal Period Reduces Postnatal Leptin Secretion in Rat and Interacts with the Effect of Protein Content in Diet
Source: PLoS One. 2013 Jul 1;8(7):e68268. doi: 10.1371/journal.pone.0068268 (PMC3698130; doi:10.1371/journal.pone.0068268)
Supplement: Table S1 — (DOCX) [file pone.0068268.s001.docx]

**Table S1.**

|  | **Control (C)** | **Control Supplemented (Csup)** | **Restricted (R)** | **Restricted Supplemented (Rsup**) |
| --- | --- | --- | --- | --- |
| Dextrose (%) | 10 | 10 | 10 | 10 |
| Sucrose (%) | 10 | 10 | 10 | 10 |
| Soybean oil (%) | 4.3 | 4.3 | 4.3 | 4.3 |
| Cellulose (%) | 5 | 5 | 5 | 5 |
| Corn starch (%) | 43.6 | 37.8 | 56.6 | 50.3 |
| Casein (%) | 22 | 22 | 9 | 9 |
| Methionine(g/kg) | 7.2 | 12 | 2.9 | 12 |
| Choline (g/kg) | 1 | 15 | 1 | 15 |
| Betaine (g/kg) | 0 | 15 | 0 | 15 |
| Vit B12 (mg/kg) | 25 | 1000 | 25 | 1000 |
| Folic acid (mg/kg) | 2 | 15 | 2 | 15 |
| Zinc (mg/kg) | 30 | 180 | 30 | 180 |
| Energy (kcal/kg) | 3260.8 | 3064.6 | 3261.6 | 3051.4 |
