# Supplementary material for: Excess of Methyl Donor in the Perinatal Period Reduces Postnatal Leptin Secretion in Rat and Interacts with the Effect of Protein Content in Diet
Source: PLoS One. 2013 Jul 1;8(7):e68268. doi: 10.1371/journal.pone.0068268 (PMC3698130; doi:10.1371/journal.pone.0068268)
Supplement: Table S2 — (DOCX) [file pone.0068268.s002.docx]

**Table S2.**

|  | **Primers for qPCR** | |
| --- | --- | --- |
| **leptin** | TTGTCACCAGGATCAATGACATTT | GACAAACTCAGAATGGGGTGAAG |
| **βactin** | CTATCGGCAATGAGCGGTTCC | GCACTGTGTTGGCATAGAGGTC |
| **B2M** | CTGGTCTTTCTACATCCTGGCT | TACATGTCTCGGTCCCAGGT |
|  | **Nested PCR primers on bisulfite converted DNA (leptin promoter)** | |
| **1^st^ PCR** | GGAAGAAGTAATTGGTTTTGGTT | AAACTCCATACCTACCTACCC |
| **2^nd^ PCR** | GGAAGAAGTAATTGGTTTTGGTT | TCTTATAATTACCCCAAT |
|  | **Sequencing primers for pyrosequencing** | |
| **CpG** | **Forward orientation** | **Reverse Orientation** |
| **1-4** | GTTTTGTAGTTGTTGG |  |
| **4-7** |  | CCTCAAAAAAAAACTTCAAC |
| **8** |  | ATAACTATTACAACCTAATACTCC |
| **9-14** | GAGTATTAGGTTGTAATAGTTAT | GACCAACCCCAATCCTTA |
| **14-16** |  | ATAATTACCCCAATACAA |
